# Elderly coagulation risk in a globalized world: burden, biomarkers, and management in Africa – a narrative review

**DOI:** 10.1097/MS9.0000000000003994

**Published:** 2025-09-30

**Authors:** Emmanuel Ifeanyi Obeagu

**Affiliations:** Department of Biomedical and Laboratory Science, Africa University, Mutare, Zimbabwe

**Keywords:** Africa, anticoagulant management, biomarkers, elderly coagulation risk, thrombotic disorders

## Abstract

The global increase in life expectancy has brought aging-related health challenges to the forefront, with coagulation disorders emerging as a significant cause of morbidity and mortality among the elderly. In Africa, the burden of coagulation abnormalities is becoming increasingly evident due to demographic shifts, the rising prevalence of noncommunicable diseases, and the persistent impact of infectious diseases that affect coagulation pathways. Despite this growing challenge, limited data and constrained healthcare resources hinder effective recognition and management of these conditions. Biomarkers such as D-dimer, prothrombin time, activated partial thromboplastin time, and platelet indices play a crucial role in diagnosing and monitoring coagulation disorders in elderly patients. However, the availability and utilization of these diagnostic tools remain inconsistent across African healthcare settings, limiting timely risk stratification and intervention. Emerging biomarkers offer potential for improved assessment.

## Introduction

The global demographic landscape is undergoing a profound transformation, with the elderly population expanding rapidly due to improved healthcare and increased life expectancy^[1,2]^. This demographic shift has brought age-related health issues, including coagulation disorders, into sharper focus. Aging is intrinsically linked with changes in hemostatic balance, increasing susceptibility to both thrombotic and hemorrhagic events^[3,4]^. These coagulation abnormalities contribute significantly to morbidity and mortality in older adults worldwide, necessitating a deeper understanding of their epidemiology, pathophysiology, and management. Africa, historically characterized by a predominantly young population, is now experiencing an unprecedented rise in its elderly demographic due to advances in healthcare and declining fertility rates. This demographic transition presents unique challenges for the continent’s healthcare systems, which have traditionally been oriented toward infectious diseases. As noncommunicable diseases (NCDs) and aging-related disorders such as coagulation abnormalities become more prevalent, African countries must adapt their clinical and public health strategies to meet these emerging needs^[5]^. The burden of coagulation risk in elderly Africans is multifactorial. Physiological changes with aging, including endothelial dysfunction, increased procoagulant factors, and reduced fibrinolytic activity, predispose older adults to thrombosis. These intrinsic factors are compounded by a high prevalence of comorbidities such as hypertension, diabetes, and chronic infections like HIV and tuberculosis, which further influence coagulation pathways. Additionally, socioeconomic disparities and healthcare infrastructure limitations exacerbate the challenges of timely diagnosis and effective management^[6–8]^.

**HIGHLIGHTS**

- Aging populations in Africa face rising thrombotic and bleeding risks due to limited awareness and healthcare infrastructure.
- Globalization increases exposure to westernized risk factors like sedentary lifestyle and processed diets.
- Biomarkers such as D-dimer, fibrinogen, and platelet indices offer promise for early detection in elderly Africans.
- Diagnostic gaps and cost barriers hinder effective coagulation disorder management across the continent.
- Integrating point-of-care testing and culturally adapted guidelines may improve outcomes in elderly coagulation risk management.

Biomarkers have a central role in identifying and stratifying coagulation risk in the elderly. Conventional tests such as D-dimer, prothrombin time (PT), activated partial thromboplastin time (aPTT), and platelet counts are widely used globally but may not be uniformly accessible or standardized across African healthcare facilities. Moreover, novel biomarkers, including thrombin generation markers and endothelial dysfunction indicators, hold promise for improving diagnostic accuracy but require validation within African populations. Developing cost-effective, reliable biomarker-based strategies suitable for local contexts is a key research priority^[9–11]^. Management of coagulation disorders in elderly Africans is challenged by factors including limited access to anticoagulant therapies, difficulties in monitoring treatment response, and sociocultural barriers to healthcare utilization. While vitamin K antagonists (VKAs) and direct oral anticoagulants (DOACs) have transformed anticoagulation therapy globally, their adoption in many African settings is constrained by cost, availability, and infrastructure. Integrating lifestyle interventions, control of comorbid conditions, and culturally appropriate patient education into management plans is necessary to improve adherence and clinical outcomes^[12–14]^. In the context of globalization, knowledge exchange and international collaboration present opportunities to address these challenges through shared research, technology transfer, and capacity building. Strengthening healthcare systems to incorporate evidence-based diagnostics and therapies, alongside community engagement and policy support, will be critical for mitigating the impact of coagulation disorders among Africa’s aging population. This review aims to synthesize current understanding of the burden, biomarkers, and management strategies of elderly coagulation risk in Africa and highlight areas for future research and intervention.

## Aim

This narrative review aims to critically examine the burden of coagulation risk among the elderly population in Africa, evaluate the current and emerging biomarkers used for diagnosis and risk stratification, and discuss context-appropriate management strategies.

## Methods

This review was conducted as a narrative synthesis aimed at exploring the burden, biomarkers, and management of coagulation risk among elderly populations in Africa within the context of globalization. To ensure a comprehensive overview, a systematic search of the literature was carried out between January 2000 and June 2025 across major electronic databases including PubMed, Scopus, Web of Science, and Google Scholar.

The following search terms and combinations were used: “elderly,” “coagulation disorders,” “thrombosis,” “bleeding,” “biomarkers,” “Africa,” “globalization,” and “anticoagulation management.” Both Medical Subject Headings (MeSH) terms and free-text words were applied to maximize sensitivity. Reference lists of relevant articles were also manually screened for additional sources.

**Inclusion criteria** were:

- Articles focusing on coagulation disorders, biomarkers, or management strategies in elderly populations.
- Studies providing African data or relevant global perspectives applicable to Africa.
- Original research, reviews, meta-analyses, and reports published in English.

**Exclusion criteria** included:

- Studies limited to pediatric or nonelderly cohorts.
- Case reports with limited generalizability.
- Non-English publications due to feasibility constraints.

All retrieved articles were screened for relevance by title and abstract, followed by full-text review. The final selection prioritized studies that provided insights into epidemiological trends, biomarker utility, and clinical or health-system management of coagulation risk in elderly populations. The extracted information was synthesized thematically under three broad domains: (1) burden of coagulation disorders, (2) biomarkers of risk, and (3) management strategies in Africa. This approach allowed integration of global perspectives with Africa-specific evidence, while highlighting existing gaps and opportunities for future research.

### Burden of elderly coagulation risk in Africa

The burden of coagulation disorders among the elderly in Africa is an emerging public health concern, fueled by demographic shifts and the increasing prevalence of age-related and chronic diseases. While the continent still faces significant challenges related to infectious diseases, the rapid growth of the aging population is bringing noncommunicable conditions, including coagulation abnormalities, into sharper focus. Thrombotic events such as venous thromboembolism (VTE), ischemic stroke, and atrial fibrillation (AF)-related complications are increasingly documented, although comprehensive epidemiological data remain limited due to underreporting and diagnostic constraints^[14]^. Several factors contribute to the heightened coagulation risk in elderly Africans. Physiological aging is associated with alterations in hemostatic balance, characterized by elevated levels of procoagulant factors like fibrinogen and factor VIII, diminished anticoagulant mechanisms, and impaired fibrinolysis. These changes predispose older individuals to a prothrombotic state. In addition, the coexistence of comorbidities common in African elderly populations, such as hypertension, diabetes mellitus, chronic kidney disease, and chronic infections (notably HIV and tuberculosis), further complicates coagulation dynamics and exacerbates risk^[15]^. The interplay between infectious diseases and coagulation abnormalities is particularly relevant in African settings. Chronic infections often trigger systemic inflammation and endothelial dysfunction, promoting hypercoagulability. For instance, HIV infection has been linked to increased risk of thrombosis through mechanisms including immune activation, altered platelet function, and coagulation cascade dysregulation. Moreover, antiretroviral therapy, while life-saving, may also impact coagulation parameters. These overlapping risk factors intensify the burden of coagulation disorders among elderly patients with chronic infectious conditions^[16]^.

Healthcare system limitations in many African countries contribute to the underdiagnosis and undertreatment of coagulation disorders. Diagnostic facilities for coagulation testing, such as D-dimer assays and advanced coagulation panels, are often unavailable or unaffordable, especially in rural and underserved areas. Furthermore, healthcare providers may have limited awareness or training in recognizing and managing thrombotic disorders in elderly patients. These factors collectively lead to delays in diagnosis and suboptimal management, increasing the risk of complications such as recurrent thromboembolism, stroke, and bleeding^[17]^. Socioeconomic factors also influence the burden of coagulation risk. Poverty, limited health insurance coverage, and geographical barriers reduce access to preventive care, diagnostics, and effective anticoagulation therapy. Cultural beliefs and low health literacy may hinder health-seeking behavior and adherence to prescribed treatments. Additionally, the high cost and limited availability of DOACs and the challenges associated with VKA monitoring further restrict optimal management in many African settings^[16,17]^.

### Biomarkers of coagulation in the elderly

Biomarkers play a pivotal role in the assessment, diagnosis, and monitoring of coagulation disorders in the elderly. Aging is associated with complex alterations in the hemostatic system, including increased procoagulant activity, impaired fibrinolysis, and endothelial dysfunction. Identifying reliable biomarkers that reflect these changes is essential for early detection of coagulation risk, risk stratification, and guiding therapeutic decisions, particularly in resource-limited settings such as many African healthcare systems^[18]^. Conventional coagulation tests remain the cornerstone of clinical evaluation. PT and aPTT are routinely used to assess the extrinsic and intrinsic coagulation pathways, respectively, providing essential information on clotting function. Elevated PT or aPTT may indicate bleeding risk or anticoagulant effect, while normal or shortened values in some contexts can reflect a hypercoagulable state. Platelet count and function tests offer additional insights, as thrombocytosis or platelet hyperactivity often accompany prothrombotic conditions in the elderly^[19]^. D-dimer, a fibrin degradation product, is widely recognized as a sensitive biomarker for the presence of ongoing coagulation and fibrinolysis. Elevated D-dimer levels are strongly associated with VTE and can serve as a screening tool to exclude thrombotic events. However, its specificity is limited, as levels can also rise in inflammatory states, infections, cancer, and with advancing age itself. This reduced specificity poses challenges in elderly African populations, where chronic infections and inflammation are prevalent^[20]^.

Emerging biomarkers offer promising avenues to improve the detection and characterization of coagulation abnormalities. Thrombin generation assays provide a dynamic measure of the balance between procoagulant and anticoagulant forces, offering superior sensitivity to conventional tests in identifying hypercoagulable states. Markers of endothelial dysfunction, such as von Willebrand factor and soluble thrombomodulin, reflect vascular injury and may predict thrombotic risk. Additionally, inflammatory markers like C-reactive protein are increasingly recognized for their role in coagulation activation, linking chronic inflammation to thrombosis in elderly patients^[21]^. Integration of biomarker data with clinical risk scores, such as the CHA2DS2-VASc score for AF or Wells’ criteria for deep vein thrombosis, enhances risk stratification accuracy. This combined approach facilitates personalized management plans that balance thrombotic and bleeding risks, especially important in elderly patients who often present with multiple comorbidities and polypharmacy. Future research focusing on validating biomarker panels in African elderly populations is essential to optimize their predictive value and clinical utility^[22]^.

## Management strategies

Effective management of coagulation risk in elderly populations requires a comprehensive, multifaceted approach that addresses prevention, early diagnosis, and appropriate therapeutic interventions. In Africa, these strategies must be adapted to local healthcare realities, including limited resources, infrastructural challenges, and sociocultural factors influencing healthcare access and adherence.

### Anticoagulant therapy

Anticoagulation remains the cornerstone of treatment for thrombotic disorders such as VTE, AF, and stroke prevention. VKAs, notably warfarin, have been widely used due to their efficacy and relatively low cost. However, VKAs require frequent monitoring of the international normalized ratio (INR) to maintain therapeutic levels, posing significant challenges in many African settings where laboratory infrastructure and patient follow-up are limited. Inconsistent monitoring increases the risk of bleeding complications or subtherapeutic anticoagulation, resulting in treatment failure^[23,24]^. DOACs represent an important advancement in anticoagulation therapy, offering fixed dosing and no routine monitoring requirements. DOACs have demonstrated favorable efficacy and safety profiles in elderly patients and, in addition, contribute to reducing the burden on healthcare systems. Despite these advantages, high costs and limited availability restrict widespread use in many African countries. Efforts to improve access through subsidy programs and inclusion in national formularies are critical to expanding DOAC use^[25]^.

### Management of comorbidities and lifestyle modifications

Addressing underlying comorbid conditions is vital to reducing coagulation risk. Hypertension, diabetes, obesity, and chronic infections such as HIV contribute to endothelial dysfunction and hypercoagulability. Effective management of these conditions through pharmacological treatment and lifestyle interventions can mitigate thrombotic risk. Encouraging physical activity, promoting healthy diets, smoking cessation, and weight management are integral components of comprehensive care, though implementing such interventions requires culturally sensitive health education tailored to local populations^[26,27]^.

### Patient education and adherence

Patient understanding and adherence to treatment regimens are crucial for successful management, particularly for elderly patients who may face cognitive decline, polypharmacy, and limited healthcare literacy. Culturally appropriate education programs that explain the risks of coagulation disorders, the importance of medication adherence, and the need for follow-up can improve outcomes. Engaging family members and caregivers is also important in supporting elderly patients through complex treatment plans^[28,29]^.

### Healthcare infrastructure and provider training

Strengthening healthcare infrastructure is fundamental to improving management of elderly coagulation risk in Africa. Expanding laboratory capacity to perform coagulation testing, ensuring the availability of essential medications, and training healthcare providers in the diagnosis and treatment of thrombotic disorders will enhance patient care. Task-shifting and continuing medical education can empower nonspecialist providers to manage anticoagulation therapy safely, particularly in rural areas^[12,30]^.

### Integration of traditional medicine

Traditional medicine remains a widely used healthcare resource in many African communities. While evidence-based management is essential, integrating traditional healers into health education and referral systems can improve early recognition and timely treatment of coagulation disorders. Collaboration between conventional healthcare providers and traditional practitioners offers a pathway to culturally acceptable and comprehensive care^[31,32]^.

### Quality-of-life measures and real-world adherence in elderly populations

The management of coagulation disorders in elderly populations extends beyond clinical outcomes such as stroke prevention or reduction of VTE. Equally important are the impacts of therapy on quality of life (QoL) and the ability of patients to adhere to long-term anticoagulation regimens in real-world settings. Elderly patients frequently present with multiple comorbidities, frailty, and cognitive or functional decline, all of which may interfere with consistent medication use and influence their perception of well-being. Polypharmacy is another common challenge, often leading to drug–drug interactions, pill burden, and confusion regarding dosing schedules, thereby undermining both adherence and overall QoL^[24,33]^. Warfarin, for many years the mainstay of anticoagulation in resource-limited settings, highlights the tension between efficacy and patient burden. Its narrow therapeutic window requires regular monitoring of the INR, frequent healthcare visits, and strict dietary considerations. These requirements impose significant physical, emotional, and financial stress on elderly patients and their caregivers. For many, anxiety about bleeding complications or fear of stroke recurrence further diminishes QoL. The unpredictability of maintaining therapeutic INR levels often compounds this distress, leading some patients to discontinue therapy altogether^[34]^.

The advent of DOACs has offered new opportunities to improve QoL among elderly populations by simplifying anticoagulation management. DOACs eliminate the need for routine laboratory monitoring and have fewer dietary restrictions, which reduces the burden on patients and healthcare systems alike. Patient-reported outcomes from international studies consistently indicate greater satisfaction and treatment convenience with DOACs compared to VKAs. However, despite these advantages, real-world adherence to DOAC therapy remains variable, particularly in elderly cohorts. Factors such as cost, limited availability in low-resource settings, and concerns over long-term safety continue to pose barriers^[35,36]^. Adherence challenges in elderly patients are influenced by both individual and systemic factors. Cognitive impairment, depression, visual decline, and limited health literacy can reduce medication-taking consistency. On a systemic level, poor access to health insurance, medication shortages, and weak follow-up systems in African healthcare contexts exacerbate these issues. Evidence suggests that while persistence with DOACs is generally higher than with warfarin, discontinuation rates remain substantial, with socioeconomic barriers often playing a decisive role in Africa^[37,38]^.

**Figure 1.**
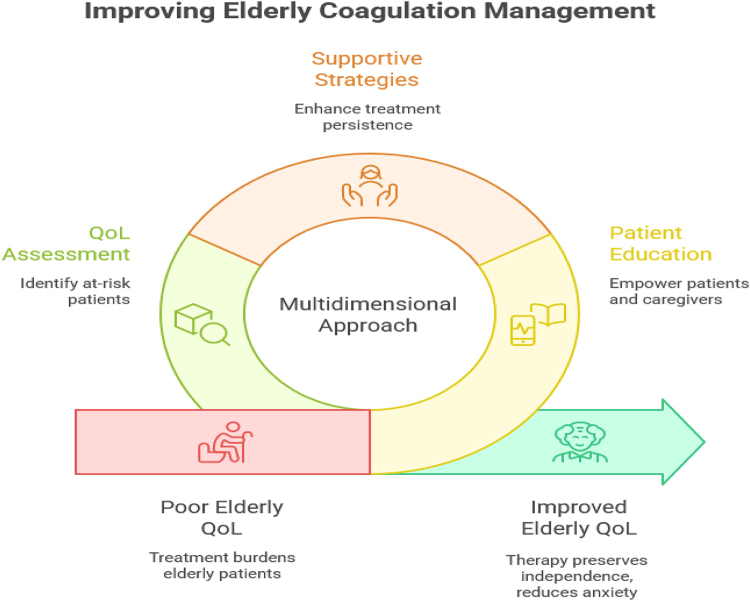
Improving elderly coagulation management.

Improving QoL and adherence requires a multidimensional approach. Incorporating patient education programs that address not only the clinical importance of anticoagulation but also practical challenges can empower patients and caregivers to engage more actively in their treatment. Supportive strategies such as family involvement, simplified dosing regimens, mobile health reminders, and community-based anticoagulation clinics can enhance treatment persistence. Furthermore, embedding QoL assessment tools – such as validated questionnaires focusing on physical, emotional, and social well-being – into routine care can help clinicians identify patients at risk of poor adherence and adjust treatment strategies accordingly^[39,40]^. Ultimately, successful management of coagulation disorders in elderly patients must balance clinical efficacy with the lived experiences of patients. Ensuring that therapy improves survival while also preserving independence, reducing anxiety, and maintaining social participation is essential. In African healthcare systems, where resource limitations intersect with cultural perceptions of illness and aging, addressing QoL and adherence represents both a challenge and an opportunity. A patient-centered, context-sensitive approach could greatly improve outcomes by aligning anticoagulation strategies with the realities of elderly patients’ daily lives (Fig. 1)^[41,42]^.

## Conclusion

The rising elderly population in Africa, coupled with complex epidemiological and healthcare challenges, underscores the growing burden of coagulation disorders on the continent. Age-related physiological changes, compounded by prevalent comorbidities and chronic infections, place older adults at heightened risk for thrombotic and bleeding complications. Despite this increasing burden, limited epidemiological data, diagnostic capacity, and access to effective therapies continue to hinder optimal care. Biomarkers remain indispensable for early detection and risk stratification of coagulation abnormalities; however, widespread application in African settings is constrained by infrastructural and economic barriers. The adoption of both conventional and emerging biomarkers tailored to local contexts is crucial for improving diagnosis and management outcomes. Management strategies must integrate anticoagulant therapy, comorbidity control, patient education, and culturally appropriate interventions within strengthened healthcare systems.

## Data Availability

Not applicable as this a Narrative Review.

## Research registration unique identifying number (UIN)

Not applicable as this a Narrative Review.
